# Prognostic value of YKL-40 in solid tumors: a meta-analysis of 41 cohort studies

**DOI:** 10.1186/s12935-019-0983-y

**Published:** 2019-10-10

**Authors:** Bingxian Bian, Li Li, Junyao Yang, Yi Liu, Guohua Xie, Yingxia Zheng, Liang Zeng, Junxiang Zeng, Lisong Shen

**Affiliations:** 1 Department of Clinical Laboratory, Xin Hua Hospital, Shanghai Jiao Tong University School of Medicine, Shanghai, China; 2 Department of Engineering, Tsinghua University, Beijing, China

**Keywords:** Prognostic value, YKL-40, Solid tumors, Meta-analysis, Overall survival

## Abstract

**Background:**

Serum/plasma YKL-40 can be a useful index that is associated with tumor development. However, the prognostic value of serum/plasma YKL-40 in patients with solid tumors is still unclear. We aimed to utilize the existing literature to investigate the prognostic value of serum/plasma YKL-40 in solid tumors.

**Methods:**

An extensive literature search for relevant studies was conducted with the Embase, Medline and Web of Science databases. The effect on survival was measured with the hazard ratio (HR). Then, pooled HRs and 95% confidence intervals (CIs) were calculated using the random and fixed-effects models according to the heterogeneity of the included studies.

**Results:**

This meta-analysis was based on 41 publications and comprised a total of 7762 patients with solid tumors. The pooled HR showed that elevated serum/plasma YKL-40 was significantly associated with poor OS (HR, 1.44; 95% CI 1.33–1.56). We also found that elevated serum/plasma YKL-40 had significant prognostic effects on OS in various cancer subgroups such as gastrointestinal tumors (HR, 1.37; 95% CI 1.18–1.58), ovarian cancer (HR, 2.27; 95% CI 1.69–3.06), melanoma (HR, 1.77; 95% CI 1.18–2.67), lung cancer (HR, 1.73; 95% CI 1.35–2.23), urologic neoplasms (HR, 1.61; 95% CI 1.08–2.40) and glioblastoma (HR, 1.23; 95% CI 1.07–1.42); in contrast, the prognostic effect of serum/plasma YKL-40 was not statistically significant in breast cancer (HR, 1.07; 95% CI 0.98–1.17).

**Conclusions:**

The available evidence supports the hypothesis that elevated serum/plasma YKL-40 is associated with poor survival in patients with solid tumors and that serum/plasma YKL-40 may serve as a novel prognostic biomarker.

## Background

There were an estimated 18.1 million new cancer cases and 9.6 million cancer deaths in 2018, and cancer is expected to rank as the leading cause of death [1]. In this setting, prognostic indicators in patients with cancer are crucial. The tumor-node-metastasis (TNM) stage is considered the prognostic indicator most strongly associated with survival. However, for patients with the same stages, while some patients have a good prognosis, and others have a poor prognosis; thus, clinical tumor staging alone cannot predict patient prognosis. Therefore, additional indicators that can be used to predict prognosis are required.

YKL-40 is a mammalian member of a chitinase protein family but contains an enzymatically inactive property [2], and it is also known as human cartilage glycoprotein-39 (hCGP-39) and chitinase-3-like-1 protein (CHI3L1). YKL-40 is secreted by a variety of cells including monocytes, neutrophils, macrophages, chondrocytes, synovial cells, and tumor cells [3]. The clear expression of YKL-40 has been reported in cancer cells [4, 5]. The exact biological functions of YKL-40 in cancer cells are still being studied. It is suggested that this protein plays a role in inflammation, stimulation of angiogenesis and regulation of extracellular tissue remodeling and thus, the expression of YKL-40 is increased in patients with cancer [6]. As a result, YKL-40 has been recognized as a new prognostic and predictive marker in many cancers. Moreover, YKL-40 plays a potential role in promoting tumor growth, which indicates that YKL-40 may serve as a therapeutic target. A mouse monoclonal anti-YKL-40 antibody (mAY) has shown to have therapeutic use in the treatment of tumor angiogenesis and metastasis [7]. The conjunctive therapy with mAY and ionizing irradiation (IR) synergistically inhibited tumor vascularization and progression in xenograft brain tumor models [8].

The first report on the prognostic value in solid tumors was a study of 41 patients with recurrent breast cancer by Johansen [9], which was followed by a series of studies that evaluated the prognostic value of serum/plasma YKL-40 in solid tumors, such as gastrointestinal tumors, ovarian cancer, melanoma, lung cancer, urologic neoplasms, glioblastoma, breast cancer, squamous cell carcinoma of the head and neck and so on. While some studies have demonstrated that serum/plasma YKL-40 has predictive and prognostic value in patients with cancer, some other studies have presented negative results. Therefore, the prognostic properties of serum/plasma YKL-40 in solid tumors remain controversial. Several meta-analyses investigated the prognostic value in certain cancers, such as glioblastoma and breast cancer [10, 11]. However, there has been no systematic analysis to quantify the existing data in solid tumors wholly. Therefore, in view of the conflicting results from previous studies, we utilized the existing literature to investigate the issue of the prognostic value of serum/plasma YKL-40 in solid tumors.

## Methods

### Search strategy

We conducted an extensive literature search for relevant studies from the Embase (from 1974 to March 08, 2019), Medline (from 1966 to March 08, 2019) and Web of Science databases (from 1985 to March 08, 2019). The search strategy included the following keywords: “YKL-40”, “YKL40”, “CHI3L1”, “chitinase-3-like-1”, “GP-39”, “glycoprotein-39”, “CGP-39”, “cartilage glycoprotein-39”, “hCGP-39”, “human cartilage glycoprotein-39”, “tumor”, “neoplasm” and “cancer”. Reports in English were eligible for inclusion. Furthermore, we manually reviewed the relevant articles to implement our search.

### Selection criteria and quality assessment

Studies were included in the meta-analysis according to the following criteria: (1) patients with a diagnosis of solid tumor that was confirmed through histopathologic examinations; (2) sufficient data were provided to determine an estimate of the hazard ratio (HR) for OS and disease-free survival (DFS)/progression-free survival (PFS); (3) more than 30 patients were enrolled in each study; and (4) cohort studies published in English. When the same patient population was used in multiple publications, only the latest was included in the meta-analysis. Reviews and comments were excluded.

Two investigators independently assessed the quality of the eligible studies. The Newcastle–Ottawa Scale (NOS) was used to assess study quality. The NOS is based on three parameters of quality: selection (0–4 points), comparability (0–2 points), and outcome assessment (0–3 points). The scores ranged from zero points (worst) to nine points (best). Disagreements on the quality assessment were resolved by discussion. We also conducted sensitivity analyses to assess the stability of the results.

### Data extraction

We extracted the following information from each study: author’s name, publication year, patients’ country, cancer type, number of patients, tumor stage, metastasis status, treatment methods, YKL-40 cutoff value, specimen type, and HR with 95% CI for DFS, PFS and OS. If the HRs and 95% CIs were not directly collected from the original article, they were calculated by the method of Parmar et al. [12].

### Statistical analysis

The pooled HR or odds ratio (OR) was initially calculated by a fixed effect model [13]. If the I^2^ statistic was more than 30% or the fixed effects P value for the I^2^ statistic was less than 0.10, indicating significant heterogeneity across studies, then a random effect model was applied to calculate the pooled HR or OR [14]. To explore the potential causes of heterogeneity, subgroup analyses and meta-regression analyses were conducted (HR was considered to be associated with covariates when the P value was less than 0.05). To evaluate the stability of the results, we conducted a sensitivity analysis by omitting each study in turn. The scope of this analysis was to evaluate the influence of a single study on the overall outcome. To investigate publication bias in the literature, we performed a visual inspection of the funnel plot symmetry and Begg’s regression and Egger’s linear regression tests (P < 0.05 was statistically significant) [15, 16]. The nonparametric trim-and-fill method was performed to further assess the potential effect of publication bias [17]. All statistical analyses were performed using the Meta package in R software (version 3.5.1, The R Project for Statistical Computing).

## Results

### Study selection procedure

**Fig. 1:**
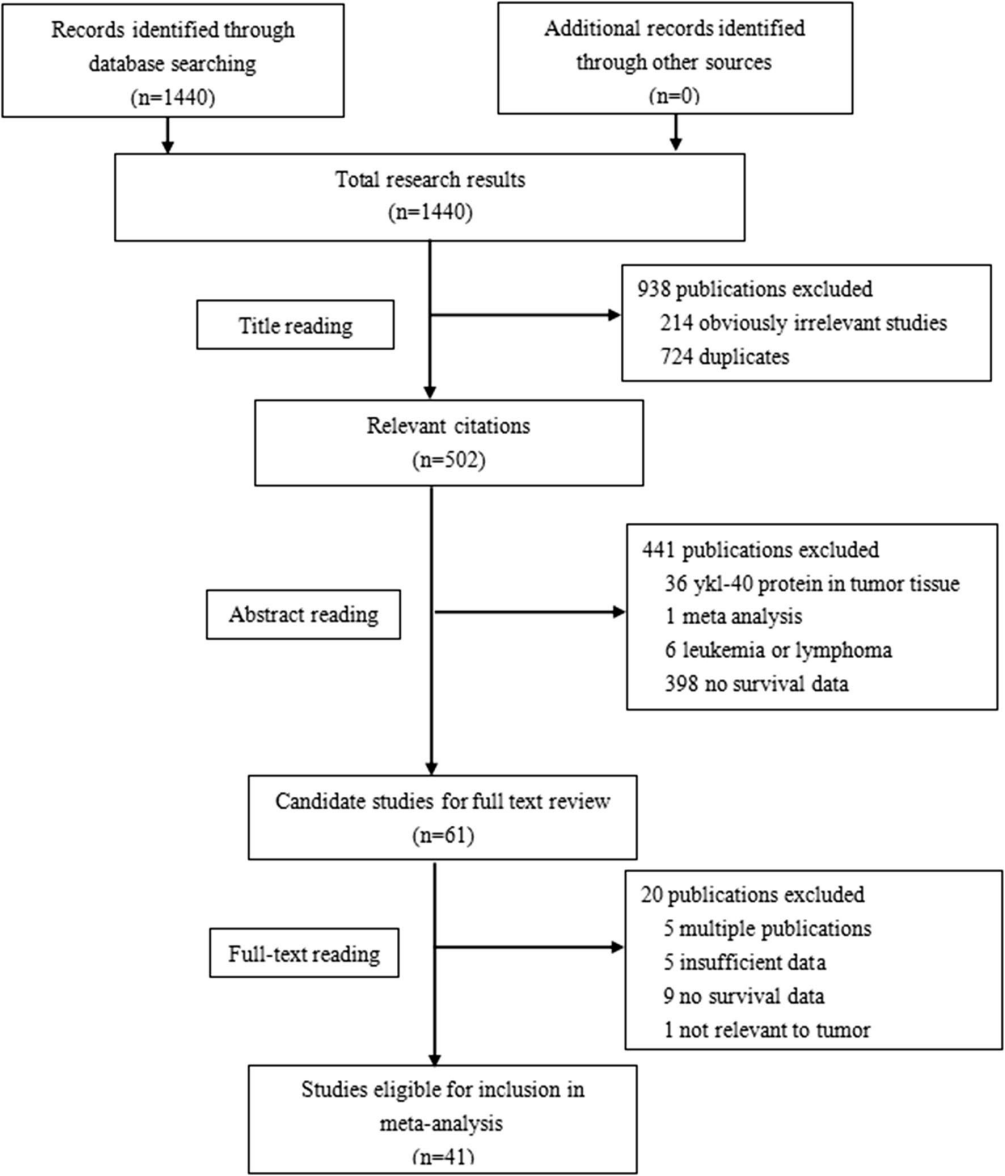
The literature search and study selection process

The flowchart of the study selection process is shown in Fig. 1. The literature search yielded a total of 1440 records. Of these articles, 502 studies were retrieved for detailed evaluation. In total, 461 studies were further excluded because of there was YKL-40 protein in the tumor tissue, the study was meta-analysis, the study involved leukemia or lymphoma, there were multiple publications, there was insufficient data, there was no survival data or the data was not relevant to tumor tissues. Finally, 41 articles [9, 18–57] met the selection criteria after reviewing the abstracts or full texts.

### Characteristics of identified studies

The characteristics of the eligible studies are summarized in Tables 1 and 2. The sample size in each study ranged from 37 to 1432 patients, and a total of 7762 patients were included in the meta-analysis. These studies were published from 1995 and 2019. 32 studies were from Caucasian countries [9, 18–32, 35–37, 41, 42, 44, 45, 47–49, 51–56], and the other 9 studies were from Asian countries [33, 34, 38–40, 43, 46, 50, 57]. The proportion of stage IV(D) or stage III(C) disease and the proportion of metastatic or extended disease were used as the proportion of advanced stage disease in 20 studies [18–20, 25, 26, 28, 29, 31, 33–35, 37, 38, 40, 43, 45, 48–50, 57] and 10 studies [21, 23, 24, 32, 44, 46, 47, 52, 54, 56], respectively. The proportion of advanced stage disease was not reported in the other 11 studies [9, 22, 27, 30, 36, 39, 41, 42, 51, 53, 55]. Johansen et al. [27] investigated the mid-therapy value of serum/plasma YKL-40, and Bernardi et al. [37] investigated the value of the serum/plasma YKL-40 ratio [(1 week value-baseline value)/baseline value]. The other studys investigated the baseline value of serum/plasma YKL-40 in solid tumors. The cutoff value of serum/plasma YKL-40 ranged from 44.6 to 247 µg/L in 24 studies [9, 18–21, 24–26, 33–36, 39, 41–43, 45, 46, 48, 50, 53–56]. In total, 6 studies [27, 30, 40, 44, 47, 51] analyzed serum/plasma YKL-40 as a continuous variable, and the cutoff value was not reported in 11 studies [22, 23, 28, 29, 31, 32, 37, 38, 49, 52, 57]. A total of 39 studies [18–54, 56, 57] reported HRs with 95% CIs for OS or DFS/PFS directly. The HR with a 95% CI for OS or DFS/PFS was estimated from Kaplan–Meier curves in the other 2 studies [9, 55]. The quality assessment of the trials is shown in Table 2.

**Table 1:** Main characteristics of the eligible studies

Study	Patients’ country	No. of patients	Tumor stage^a^	Treatment methods	Cutoff value	Specimen type
Breast cancer						
Johansen 1995 [9]	Denmark	41	–	Chemotherapy	207 μg/L	Serum
Jensen 2003 [21]	Denmark	100	Metastatic disease (100)	Chemotherapy	168 μg/L	Serum
Johansen 2003 [22]	England	271	–	Surgery and chemotherapy and radiotherapy	–	Serum
Yamac 2008 [29]	Turkey	45	I–III (62.2)	Surgery and chemotherapy	–	Serum
Wang 2012 [38]	China	120	TNM I–III (23.3)	Surgery	–	Serum
Gastrointestinal tumors						
Cintin 1999 [18]	Denmark	603	Dukes’ staging A–D (53.4)	Surgery	247 μg/L	Serum
Chang 2009 [30]	America	52	II–III (–)	Chemotherapy and radiotherapy	Continuous variable	Plasma
Zhu 2012 [39]	China	212	–	Chemoembolization	106 μg/L	Serum
Zhu 2012 [40]	China	158	TNM I–IV (38.0)	Surgery	Continuous variable	Serum
Schultz 2013 [41]	Denmark and Germany	103	–	Surgery; chemotherapy (not undergoing surgery)	116 μg/L	Plasma
		370				
Liu 2014 [43]	China	86	UICC I–IV (44.2)	Surgery and chemotherapy	216 μg/L	Serum
Tarpgaard 2014 [44]	32 Nordic centers	510	Metastatic disease (100)	Chemotherapy	Continuous variable	Plasma
Jensen 2016 [47]	Denmark	162	Metastatic disease (100)	Cetuximab and irinotecan	Continuous variable	Plasma
		98				
Thongsom 2016 [50]	Thailand	57	TNM I–IV (78.9)	Surgery	100.7 μg/L	Plasma
Gramkow 2017 [52]	Finland	457	Metastatic disease (100)	Liver resection	–	Serum
Fuksiewicz 2018 [55]	Poland	83	–	Surgery	44.6 μg/L	Serum
Ovarian cancer						
Dehn 2003 [19]	Denmark	73	I–IV (75.3)	Chemotherapy	160 μg/L	Plasma
Høgdall 2003 [20]	Denmark	47	III(100)	Surgery	130 μg/L	Plasma
Høgdall 2009 [31]	Denmark	76	I–IV (72.4)	Surgery and chemotherapy	–	Plasma
Boisen 2016 [49]	Denmark	140	FIGO I–IV (82.1)	Bevacizumab	–	Plasma
Lung cancer						
Johansen 2004 [23]	Denmark	131	Limited and extended disease (55.0)	Chemotherapy	–	Serum
Choi 2010 [34]	Korea	39	IIIB–IV (100)	Chemotherapy	165 μg/L	Serum
Thöm 2010 [35]	Germany	189	III–IV (100)	Chemotherapy	209 μg/L	Serum
Xu 2014 [46]	China	120	Limited and extended disease (41.7)	Chemotherapy	65.7 μg/L	Serum
Matsuo 2019 [57]	Japan	50	III–IV (100)	Anti-PD-1 inhibitor	–	Plasma
Urologic neoplasms						
Brasso 2006 [24]	Denmark	152	Metastatic disease (100)	Endocrine therapy	104 μg/L	Serum
Johansen 2007 [27]	Denmark	102	–	Total androgen ablation or parenteral estrogen	Continuous variable	Serum
Tschirdewahn 2014 [45]	Germany	101	T stage T_a_–T_4_ (45.5)	Surgery	90 μg/L	Serum
Vom Dorp 2016 [48]	Germany	152	Stage pT1–T4 (40.1)	Surgery	185 μg/L	Serum
Väänänen 2017 [54]	Finland	82	Metastatic and non-metastatic disease (25.6)	Surgery	120 μg/L	Serum
Darr 2018 [56]	Germany	109	Metastatic disease(100)	Chemotherapy	160 μg/L	Serum
Melanoma						
Schmidt 2006 [25]	Denmark	225	I–II (0)	Surgery	124 μg/L	Serum
Schmidt 2006 [26]	Denmark	110	IV (100)	Chemotherapy and immunotherapy	124 μg/L	Serum
Krogh 2016 [51]	Europe	299	Stage IIB and III (–)	Untreated	Continuous variable	Serum
Erturk 2017 [53]	Turkey	112	–	chemotherapy and radiotherapy and immunotherapy	174.88 μgL	Serum
Squamous cell carcinoma of the head and neck						
Roslind 2008 [28]	Denmark	144	I–IV (55.6)	Radiotherapy	–	Serum
Multiple tumors						
Johansen 2009 [32]	Denmark	1432	Localized disease and metastatic disease (40.8)	–	–	Plasma
Cervical adenocarcinoma						
Mitsuhashi 2009 [33]	Japan	37	I–IV (29.7)	Surgery and chemoradiation	130 μg/L	Serum
Glioblastoma						
Iwamoto 2011 [36]	America	141	–	Surgery	98 μg/L	Serum
Bernardi 2012 [37]	Italy	60	Astrocytoma Grade IV (100)	Surgery and irradiation and chemotherapy	–	Serum
Gállego 2014 [42]	France	111	–	Surgery	60 μg/L	Plasma

– not reported

^a^Tumor stage and proportion of advanced stage(%)

**Table 2:** Main results

Author	Outcome	HR	95% CI	NOS score
Breast cancer				
Johansen 1995	OS	2.2	0.83–5.81	7
Jensen 2003	OS	2.57	1.6–4.1	9
	PFS	1.96	1.2–3.1	
Johansen 2003	OS	1.77	1.03–3.06	8
Yamac 2008	OS	1.004	1.00–1.07	7
Wang 2012	OS	1.04	1.02–1.06	7
	DFS	1.02	1.00–1.03	
Gastrointestinal tumors				
Cintin 1999	OS	1.4	1.1–1.8	8
Chang 2009	OS	0.99	0.76–1.28	7
Zhu 2012	OS	1.809	1.259–2.601	8
Zhu 2012	OS	2.188	1.237–3.870	8
Schultz 2013	OS	0.69	0.36–1.33	7
	OS	1.30	1.03–1.64	
Liu 2014	PFS	1.653	1.123–2.416	7
Tarpgaard 2014	OS	1.17	1.05–1.30	7
	PFS	1.00	0.91–1.09	
Jensen 2016	OS	1.53	1.1–2.13	6
	OS	2.89	1.84–4.53	
Thongsom 2016	OS	1.642	0.780–3.455	7
Gramkow 2017	OS	1.19	1.05–1.34	6
Fuksiewicz 2018	OS	1.5	0.36–6.2	7
	DFS	0.93	0.39–2.24	
Ovarian cancer				
Dehn 2003	OS	2.27	1.27–4.06	7
Høgdall 2003	OS	3.95	1.52–10.273	7
Høgdall 2009	OS	2.13	1.40–3.25	7
Boisen 2016	OS	1.97	0.90–4.32	7
	PFS	2.91	1.07–7.92	
Lung cancer				
Johansen 2004	OS	1.96	1.13–3.40	7
Choi 2010	OS	3.6	1.25–10.39	7
Thöm 2010	OS	1.48	1.04–2.10	8
Xu 2014	OS	1.84	1.08–3.15	7
	PFS	1.12	1.01–1.23	
Matsuo 2019	PFS	1.119	0.992–1.262	7
Urologic neoplasms				
Brasso 2006	OS	1.3	1.0–1.7	8
Johansen 2007	OS	1.0	0.7–1.3	7
Tschirdewahn 2014	OS	1.837	1.039–3.375	8
Vom Dorp 2016	OS	3.854	2.222–6.686	8
Väänänen 2017	OS	3.19	1.38–7.36	7
Darr 2018	OS	0.933	0.621–1.401	6
Melanoma				
Schmidt 2006	OS	3.6	1.7–7.7	9
Schmidt 2006	OS	1.9	1.2–2.8	9
Krogh 2016	OS	1.28	1.05–1.57	8
Erturk 2017	OS	1.568	0.580–3.051	7
Squamous cell carcinoma of the head and neck				
Roslind 2008	OS	2.16	1.39–3.35	9
Multiple tumors				
Johansen 2009	OS	1.8	1.3–2.5	9
Cervical adenocarcinoma				
Mitsuhashi 2009	DFS	11	1.29–97	8
Glioblastoma				
Iwamoto 2011	OS	1.2	1.0–1.4	8
Bernardi 2012	OS	1.97	1.03–3.8	6
Gállego 2014	OS	1.21	0.89–1.64	7
	PFS	1.09	0.83–1.42	

*HR* hazard ratio, *CI* confidence interval, *NOS* Newcastle–Ottawa scale, *OS* overall survival, *PFS* progression-free survival, *DFS* disease-free survival

### Effect of the value of serum/plasma YKL-40 on OS in solid tumors

**Fig. 2:**
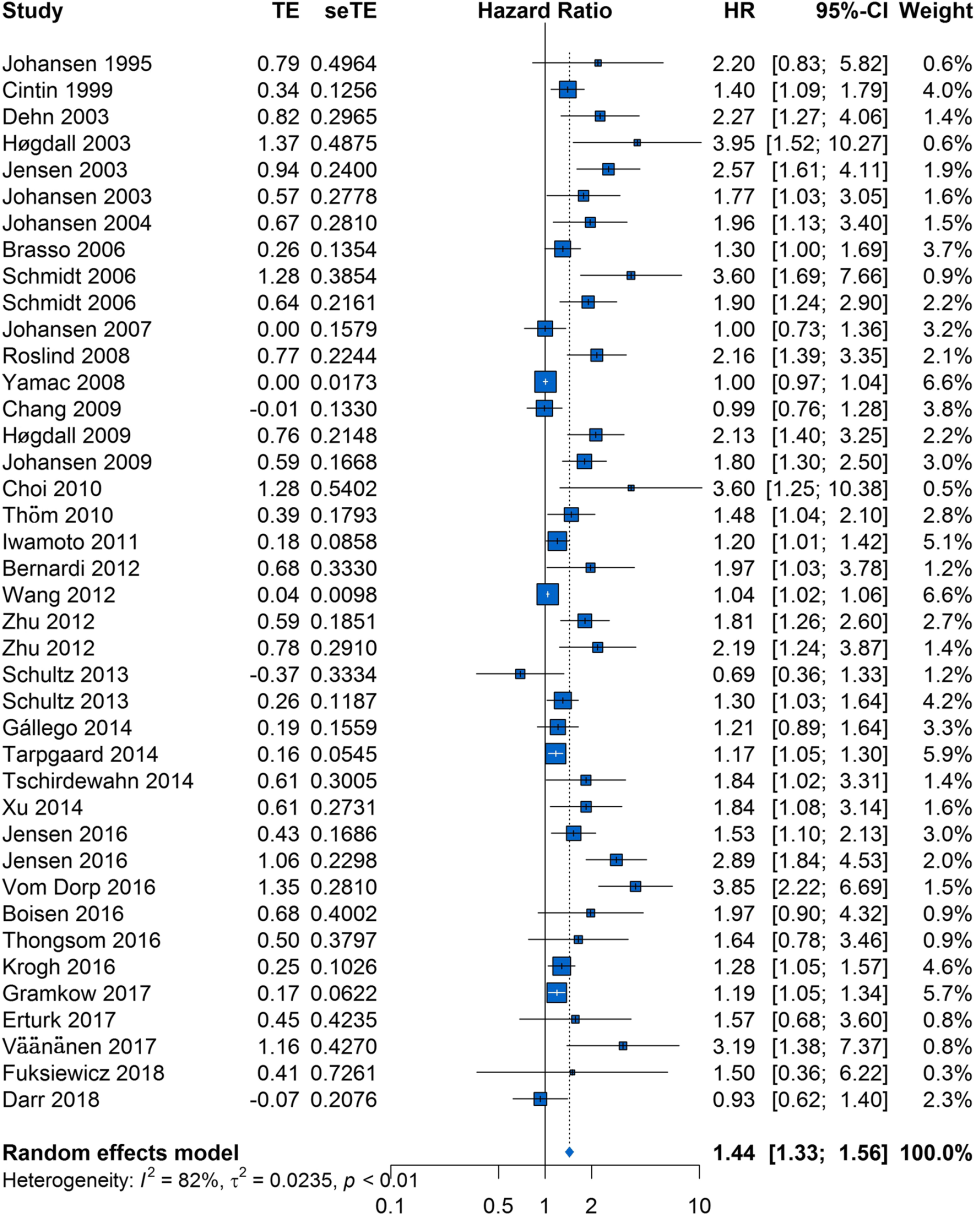
Forest plot showing the meta-analysis of the hazard ratio estimates for overall survival in all patients

The HRs for OS were available in 38 studies [9, 18–32, 34–42, 44–56], and 2 HRs were extracted from 2 studies each because 2 cohorts were used in these studies. The forest plot of all studies is provided in Fig. 2. As heterogeneity among studies clearly existed (P < 0.01, I^2^ = 82%), a random-effects model was applied. The pooled HR showed that elevated serum/plasma YKL-40 was significantly associated with poor OS (HR, 1.44; 95% CI 1.33–1.56).

To explore potential causes of heterogeneity, we performed meta-regression analyses using the following covariates: ethnicity, publication year, YKL-40 cutoff value, sample size, proportion of advanced stage disease, and specimen type, and treatment method(surgery vs. other treatment methods). The results indicated that ethnicity (P = 0.5611; Table 3), publication year (P = 0.4102), YKL-40 cutoff value (P = 0.5199), sample size (P = 0.3790), proportion of advanced stage disease (P = 0.2221), specimen type (P = 0.9164) and treatment method (0.7215) did not contribute to the cause of heterogeneity.

**Table 3:** Results of meta-regression analyses exploring causes of heterogeneity with overall survival in solid tumors

Covariates	OS Multivariate analysis
	P
Ethnicity	0.5611
Publication year	0.4102
YKL-40 cutoff value	0.5199
Sample size	0.3790
Proportion of advanced stage	0.2221
Specimen type	0.9164
Treatment method	0.7215

*OS* overall survival

**Fig. 3:**
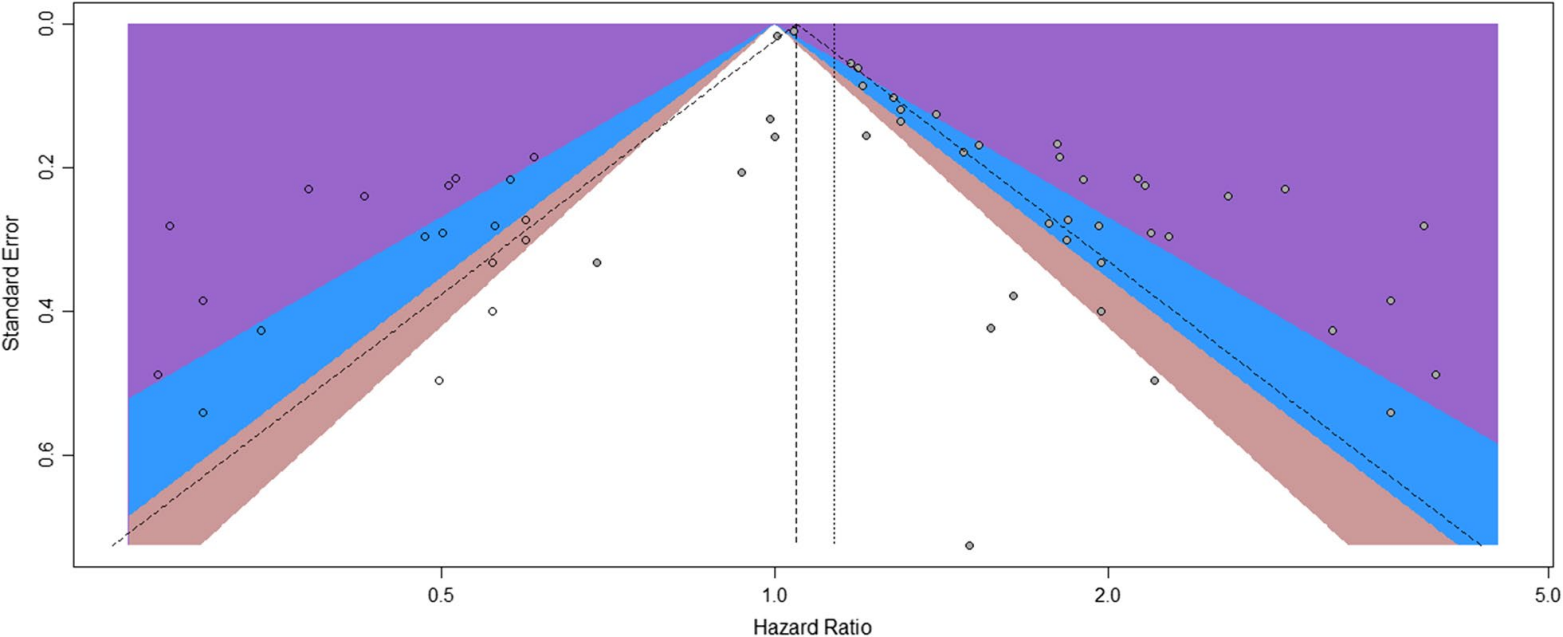
Contour-enhanced funnel plot of the association between serum/plasma YKL-40 and overall survival in all patients. The hollow circles represent the missing studies that the trim-and-fill method identified

A visual inspection of the funnel plot revealed asymmetry. This was confirmed by Egger’s test (P < 0.01), although Begg’s test did not indicate statistical significance (P = 0.244). For this reason, we performed a trim-and-fill analysis and found that 19 studies might be missing (Fig. 3). When these studies were published, the adjusted HR was 1.13 (95% CI 1.05–1.22; P < 0.01, I^2^ = 85%; Additional file 1: Figure S1), and the results continued to show a statistically significant association between serum/plasma YKL-40 and OS. The sensitivity analysis indicated that no individual studies significantly affected the overall outcome and demonstrated the stability of the results (Additional file 2: Figure S2).

### Effect of the value of serum/plasma YKL-40 on OS in gastrointestinal tumors

**Fig. 4:**
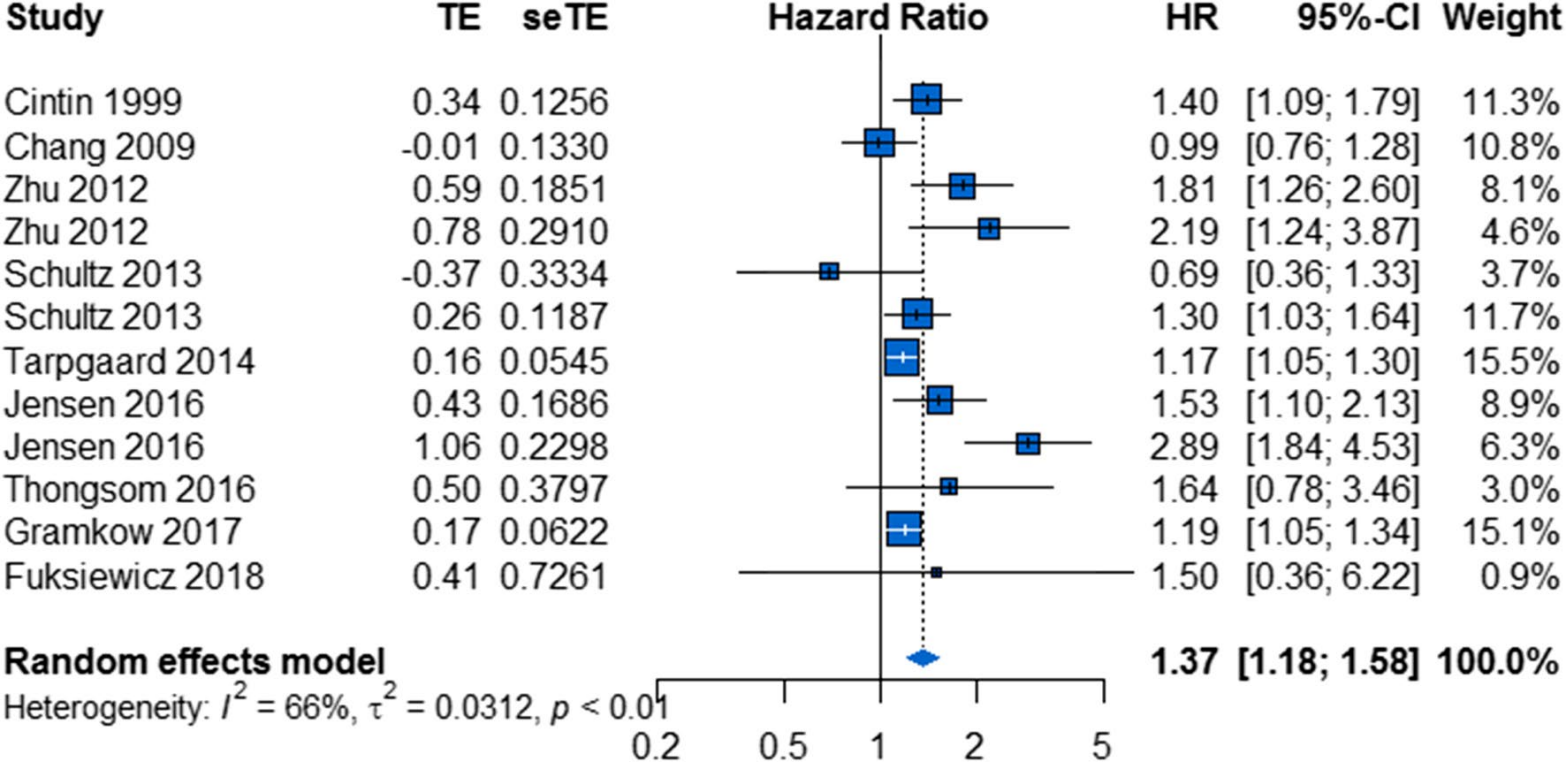
Forest plot showing the meta-analysis of hazard ratio estimates for overall survival in gastrointestinal tumors

A total of 10 studies comprising 2865 patients reported 12 HRs for OS in gastrointestinal tumors [18, 30, 39–41, 44, 47, 50, 52, 55]. Overall, elevated serum/plasma YKL-40 was associated with poor OS (HR, 1.37; 95% CI 1.18–1.58; Fig. 4). As heterogeneity existed among studies (P < 0.01, I^2^ = 66%), a random-effects model was applied.

In the subgroup analysis based on ethnicity, there was a significant relationship between elevated serum/plasma YKL-40 and poor OS in both the “Caucasian countries” subgroup and the “Asian countries” subgroup (HR, 1.28; 95% CI 1.11–1.48 and HR, 1.87; 95% CI 1.41–2.48, respectively; Additional file 3: Figure S3a). Statistical heterogeneity was significant in the “Caucasian countries” subgroup, whereas it was not significant in the “Asian countries” subgroup (I^2^ = 66%, P < 0.01 and I^2^ = 0%, P = 0.80, respectively).

In addition, we investigated the prognostic role of serum/plasma YKL-40 with respect to OS for patients who received different treatment methods. Patients received surgery alone in some studies and other treatment methods in the other studies. There was a significant relationship between elevated serum/plasma YKL-40 and poor OS in both the “surgery” subgroup and the “other treatment methods” subgroup (HR, 1.31; 95% CI 1.05–1.63 and HR, 1.43; 95% CI 1.14–1.80, respectively; Additional file 3: Figure S3b). Statistical heterogeneity was significant in the “other treatment methods” subgroup, whereas it was not significant in the “surgery” subgroup (I^2^ = 79%, P < 0.01 and I^2^ = 44%, P = 0.11, respectively).

To explore potential causes of heterogeneity, we performed meta-regression analyses using the following covariates: ethnicity, publication year, YKL-40 cutoff value, sample size, proportion of advanced stage disease, specimen type, and treatment method. The results indicated that the only explanatory variable that influenced HR was ethnicity (P = 0.0407, Table 4) and that publication year (P = 0.5750), YKL-40 cutoff value (P = 0.0908), sample size (P = 0.6562), proportion of advanced stage disease (P = 0.4457), specimen type (P = 0.4700), and treatment method (P = 0.6596) did not contribute to the cause of heterogeneity.

**Table 4:** Results of meta-regression analyses exploring causes of heterogeneity with overall survival in gastrointestinal tumor

Covariates	OS Univariate analysis
	P
Ethnicity	0.0407
Publication year	0.5750
YKL-40 cutoff value	0.0908
Sample size	0.6562
Proportion of advanced stage	0.4457
Specimen type	0.4700
Treatment method	0.6596

*OS* overall survival

A visual inspection of the funnel plot did not reveal asymmetry (Additional file 4: Figure S4). This was confirmed by Egger’s test (P = 0.1129) and Begg’s test (P = 0.337). The sensitivity analysis indicated that no individual studies significantly affected the overall outcomes and demonstrated the stability of the results (Additional file 5: Figure S5).

### Effect of the value of serum/plasma YKL-40 on OS in other cancers

**Fig. 5:**
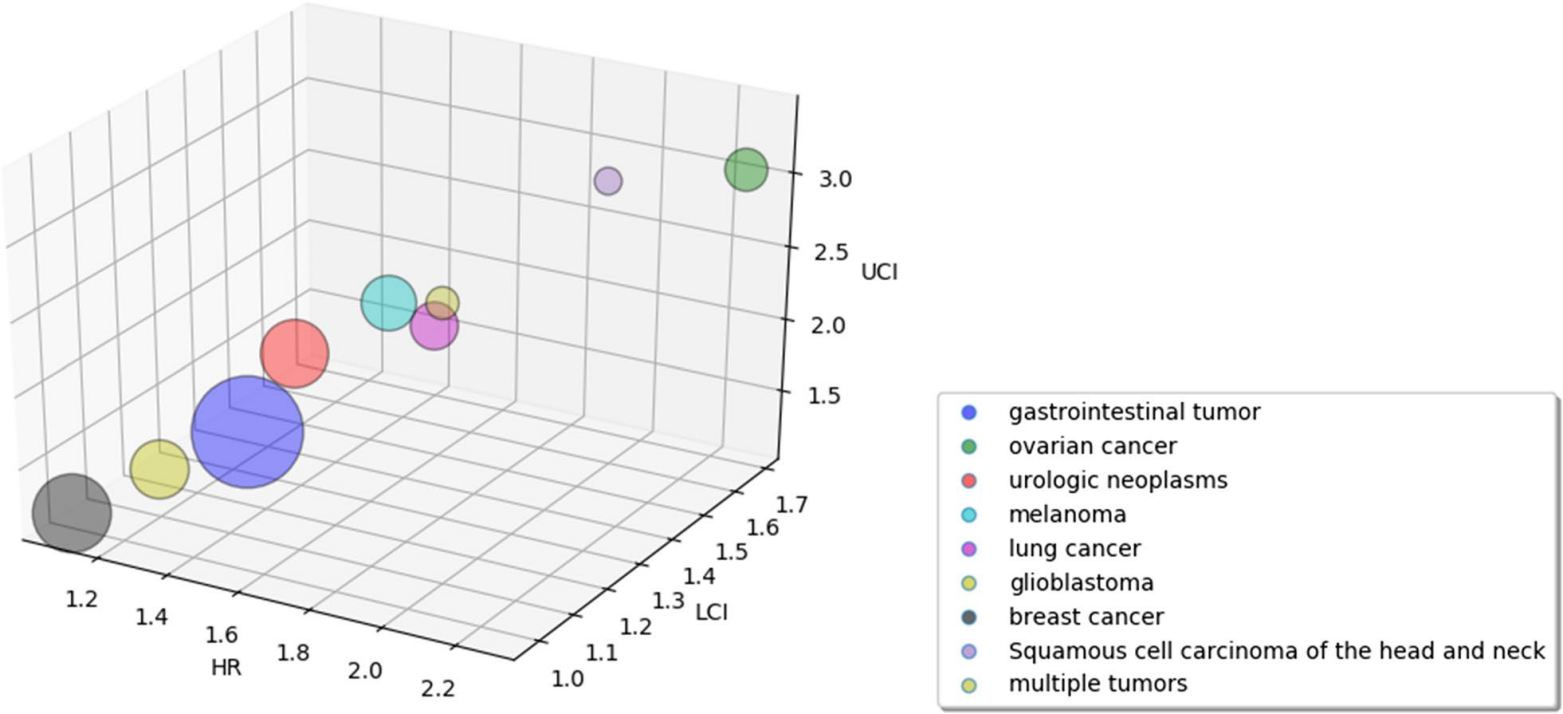
Hazard ratios and 95% confidence intervals by disease subgroups. The x-axis represents the hazard ratio for each subgroup, the y-axis represents the LCI, and the z-axis represents the UCI. *LCI* lower confidence interval, *UCI* upper confidence interval

**Fig. 6:**
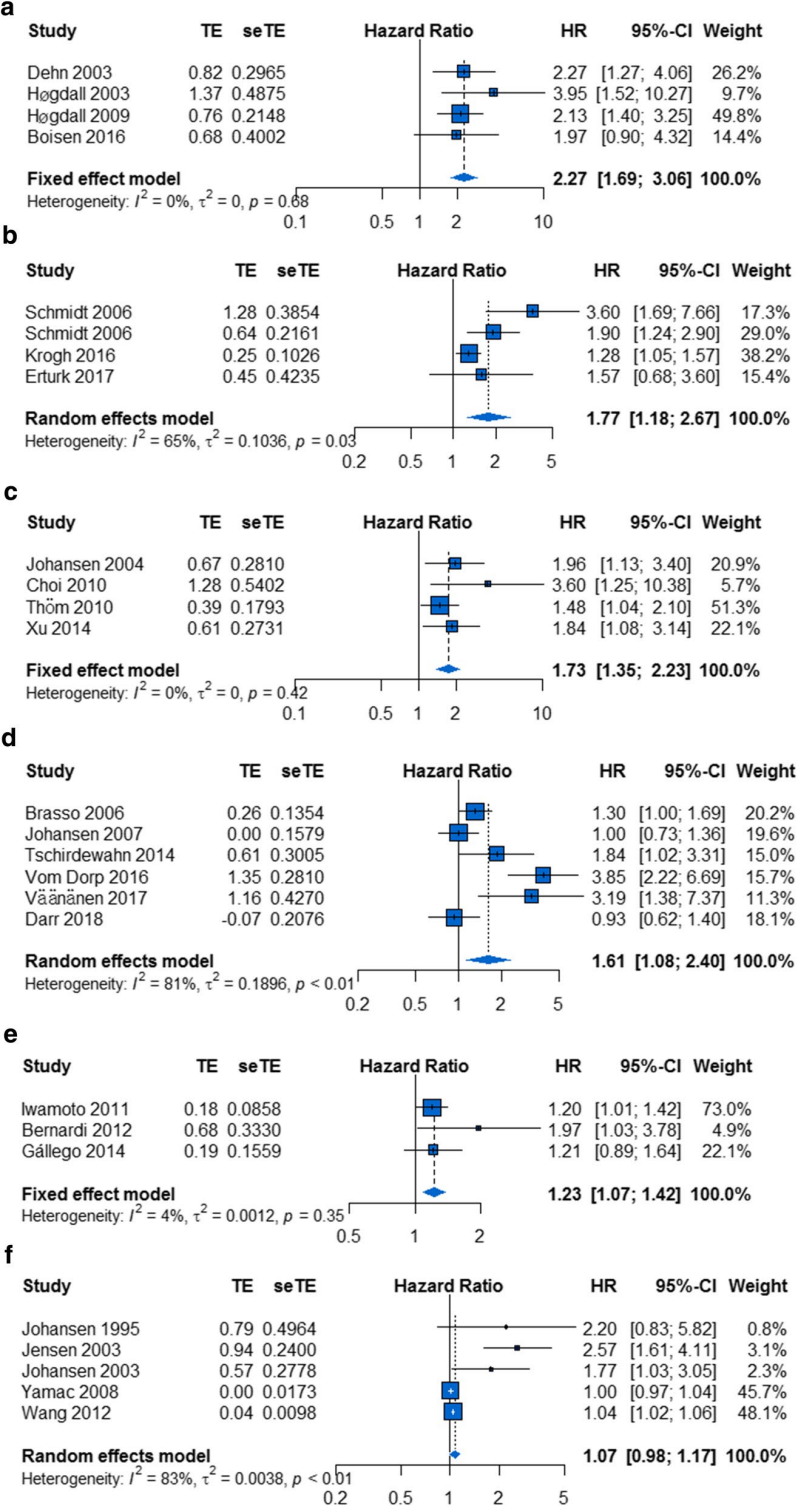
Forest plot showing the meta-analysis of hazard ratio estimates for overall survival in **a** ovarian cancer, **b** melanoma, **c** lung cancer, **d** urologic neoplasms, **e** glioblastoma, and **f** breast cancer

As shown in Fig. 5, the prognostic effect of serum/plasma YKL-40 was highest in ovarian cancer (HR, 2.27; 95% CI 1.69–3.06; P = 0.68, I^2^ = 0%; Fig. 6a), followed by melanoma (HR, 1.77; 95% CI 1.18–2.67; P = 0.03, I^2^ = 65%; Fig. 6b), lung cancer (HR, 1.73; 95% CI 1.35–2.23; P = 0.42, I^2^ = 0%; Fig. 6c), urologic neoplasms (HR, 1.61; 95% CI 1.08–2.40; P < 0.01, I^2^ = 81%; Fig. 6d) and glioblastoma (HR, 1.23; 95% CI 1.07–1.42; P = 0.35, I^2^ = 4%; Fig. 6e); in contrast, the prognostic effect of serum/plasma YKL-40 was not statistically significant in breast cancer (HR, 1.07; 95% CI 0.98–1.17; P < 0.01, I^2^ = 83%; Fig. 6f).

### Effect of the value of serum/plasma YKL-40 on DFS/PFS in solid tumors

In total, 10 HRs for DFS/PFS were available in 10 studies [21, 33, 38, 42–44, 46, 49, 55, 57]. The estimated pooled HR for these studies showed a high risk of disease progression in patients with elevated serum/plasma YKL-40 (HR, 1.11; 95% CI 1.02–1.22; Additional file 6: Figure S6). As heterogeneity existed among studies (P < 0.01, I^2^ = 68%), a random-effects model was applied.

To explore potential causes of heterogeneity, we performed meta-regression analyses using the following covariates: ethnicity, publication year, YKL-40 cutoff value, sample size, proportion of advanced stage disease, and specimen type and treatment method. The results indicated that the only explanatory variable that influenced HR was the YKL-40 cutoff value (P = 0.0017; Additional file 7: Table S1) and that ethnicity (P = 0.9445), publication year (P = 0.6929), sample size (P = 0.0538), proportion of advanced stage disease (P = 0.8162), and specimen type (P = 0.4427) and treatment method (P = 0.3932) did not contribute to the cause of heterogeneity.

### YKL-40 and clinicopathological parameters

Several studies investigated the associations between YKL-40 and clinicopathological parameters. Eight studies reported related data. Of these, 4 studies reported the association between YKL-40 and tumor stage (III-IVvs.I-II, C-D vs. A-B or extended vs. limited); 3 studies reported the association between YKL-40 and metastasis status (lymph node or liver metastasis vs. no metastasis); and 1 study reported both. Pooled outcome from five studies demonstrated a strong association between YKL-40 and clinical stage(OR, 1.47; 95% CI 1.02–2.12; Additional file 8: Figure S7a). Unfortunately, similar association was not observed between YKL-40 and metastasis status (OR, 2.14; 95% CI 0.89–5.14; Additional file 8: Figure S7b) in 4 studies.

## Discussion

YKL-40 has been suggested to have prognostic value in various cancers. Previous studies showed the prognostic value of serum/plasma YKL-40 in solid tumors was controversial and it was lack of high quality study. Here, we performed a meta-analysis of 41 studies comprising 7762 patients with solid tumors to evaluate the prognostic value of YKL-40. To our acknowledge, several meta-analyses investigated the prognostic value in certain cancers, such as glioblastoma and breast cancer [10, 11]. However, our study is the first systematic analysis to quantify the existing data in solid tumors wholly.

Reportedly, Jeet’s study revealed that knockdown of YKL-40 in the bone metastatic C4-2B cells decreased both migration and invasion, whereas overexpression in less aggressive LNCaP cells rendered them more migratory and invasive [58]. Moreover, Ku’s study showed that YKL-40 affected glioma cell invasion through regulation of MMP-2 expression, adhesion to ECM, cytoskeleton rearrangement and contractility [5]. In addition, YKL-40 significantly promoted the chemotaxis of macrophages and the angiogenesis accompanied by the increased secretion of IL-8 and MCP-1 through the MAPK signaling pathway [4]. Based on these findings, YKL-40 was thought to be a prognostic and predictive marker in tumors. In our study, elevated serum/plasma YKL-40 was significantly associated with poor OS (HR, 1.44; 95% CI 1.33–1.56), which meant serum/plasma YKL-40 could be a prognostic marker in solid tumors and confirmed the above point. Moreover, our meta-analysis also confirmed that YKL-40 was closely associated with clinical stage, which indicated that elevated YKL-40 may promote advanced stage because of its biology role, such as angiogenesis [6]. However, we failed to discover the similar result with regard to metastasis, which may be caused by the small sample size included.

We also found significant prognostic effects of elevated serum/plasma YKL-40 on OS among various cancer subgroups, such as gastrointestinal tumors, ovarian cancer, melanoma, lung cancer, urologic neoplasms and glioblastoma; in contrast, the prognostic effect of serum/plasma YKL-40 was not significant in breast cancer. Wan’s study showed elevated YKL-40 expression was significantly associated with poor overall survival in breast cancer [11], and it seems that our results are not the same as its results. That is probably largely because of selection criteria. Unlike us, both the correlation between serum/plasma YKL-40 and prognosis and the correlation between YKL-40 in tissue and prognosis were chosen to study in Wan’s study [11], and thus our 95% CI of HR for the prognostic value of YKL-40 in breast cancer would be expanded because of the smaller sample size. To a large extent, it led to our negative result. In view of this, the prognostic value of serum/plasma YKL-40 in breast cancer still needs to be assessed through large studies.

Clinically, the expression of YKL-40 has been observed in serum/plasma and tissue. For example, several studies investigated the prognostic value of YKL-40 in tissue in various tumors, such as glioblastoma [59, 60] and breast cancer [61, 62]. However, some patients with tumors may not require surgeries and unnecessary tumor biopsies carry some risks for patients. Therefore, serum/plasma YKL-40 is more promising. Moreover, a study of 10-year period in healthy subjects showed plasma had minimal intraindividual variability [63].

Plasma and serum YKL-40 levels were both used in our included studies. Certain tests may require a certain specimen for the measurement, but the meta-regression results showed that the specimen type did not influence the HR, which means that the prognostic value of YKL-40 was not influenced by the specimen type.

Different YKL-40 cutoff values were used in our included studies. Although some studies used the 95% percentile of the serum YKL-40 concentration in healthy controls, the selection criteria of some studies were still unclear. Although the YKL-40 cutoff value may influence the HR for DFS/PFS according to the meta-regression in solid tumors, it was unlikely to influence the overall prognostic value of YKL-40 in various cancers because YKL-40 cutoff value did not influence the HR for OS according to the meta-regression in solid tumors. More large studies are needed to evaluate the optimal YKL-40 cutoff value for prognostic assessment in solid tumors.

Subgroup analysis by ethnicity in gastrointestinal tumors indicated that statistical heterogeneity was found in the “Caucasian countries” subgroup, whereas it was not significant in the “Asian countries” subgroup (I^2^ = 66%, P < 0.01 and I^2^ = 0%, P = 0.80, respectively). We also found that ethnicity (P = 0.0407) could influence HR in the meta-regression. This result means that ethnicity was another potential source of heterogeneity, in part because of the low number of studies about Asian populations and discrepancies between studies. More large studies are needed to assess the prognostic value of YKL-40 in gastrointestinal tumors in Asian populations.

In addition, the subgroup analysis by treatment method in gastrointestinal tumors suggested that the prognostic value of serum/plasma YKL-40 for OS was significant in both the “surgery” subgroup and the “other treatment methods” subgroup. However, it is difficult to determine whether the prognostic effect of YKL-40 was independent of treatment methods based on available studies. The reason for this drawback is that there was marked heterogeneity in the patients’ response to different treatment regimens. Further studies are needed to evaluate the relevant prognostic factors.

The present study has several advantages. First, we performed a comprehensive and systematic search for relevant studies without limitations on the country of origin. Second, a meta-analysis with 8 included studies was performed to investigate the prognostic value of YKL-40 in glioblastoma by Qin et al. [10], and another meta-analysis with 10 included studies was performed to investigate the prognostic value of YKL-40 in breast cancer by Wan et al. [11] Both found that elevated YKL-40 was associated with poor prognosis. Our meta-analysis included 41 cohort studies and 7762 patients and thus is larger than the previous studies, which could lead to an increase in the statistical power and more precisely evaluate the prognostic value of YKL-40 in solid tumors. Third, the type of tumors was broadly defined, and the studies included gastrointestinal tumors, ovarian cancer, urologic neoplasms, melanoma, lung cancer, glioblastoma and breast cancer. Therefore, our meta-analysis performed a large-scale investigation of the existing data in solid tumors wholly. Fourth, subgroup analyses and meta-regression analyses were conducted to explore the potential causes of heterogeneity such as ethnicity, publication year, YKL-40 cutoff value, sample size, proportion of advanced stage disease, specimen type and treatment method; we found that the YKL-40 cutoff value and ethnicity may influence the HR according to the meta-regression.

There are limitations to this meta-analysis. First, we found publication bias, which indicated that fewer negative results were published than would be expected. Although we tried our best to conduct an extensive literary search for relevant studies, it is inevitable that some studies were missing. However, after performing a trim-and-fill analysis, we found that even when the 19 missing studies were published, an elevated serum/plasma YKL-40 was still associated with poor OS in solid tumors. Second, the number of studies was not sufficient for Asian populations. Third, our study is based on summarized data, and we did not obtain updated individual patient data, which may reduce the accuracy of the results. Fourth, significant heterogeneity existed among the studies. Although the YKL-40 cutoff value and ethnicity influenced HR in the meta-regression, different experimental designs, individual treatment regimens and lifestyles may also contribute to the heterogeneity. Therefore, further large multicenter prospective studies based on homogeneous populations should be conducted.

## Conclusion

In conclusion, the available evidence supports the hypothesis that elevated serum/plasma YKL-40 is associated with poor survival in patients with solid tumors and that YKL-40 may serve as a novel prognostic biomarker. We also found significant prognostic effects of elevated serum/plasma YKL-40 on OS in various cancer subgroups such as gastrointestinal tumors, ovarian cancer, melanoma, lung cancer, urologic neoplasms and glioblastoma, whereas the prognostic effect of serum/plasma YKL-40 was not statistically significant in breast cancer. Therefore, further large prospective studies using standardized unbiased methods still should be conducted to assess the prognostic effect of serum/plasma YKL-40 in breast cancer.

## Supplementary information

**Additional file 1: Figure S1.** Forest plot showing the meta-analysis of hazard ratio estimates for overall survival in all patients after the trim-and-fill method was applied.

**Additional file 2: Figure S2.** Sensitivity analysis for the pooled hazard ratios in overall survival in all patients. The analysis was conducted by estimating the average hazard ratio in the absence of each study.

**Additional file 3: Figure S3.** Forest plot showing the meta-analysis of hazard ratio estimates for overall survival in (a) the “Caucasian countries” subgroup and the “Asian countries” subgroup and (b) the “surgery” subgroup and “other treatment methods” subgroup.

**Additional file 4: Figure S4.** Contour-enhanced funnel plot of the association between serum/plasma YKL-40 and overall survival in gastrointestinal tumors.

**Additional file 5: Figure S5.** Sensitivity analysis for the pooled hazard ratios in all patients with gastrointestinal tumors. The analysis was conducted by estimating the average hazard ratio in the absence of each study.

**Additional file 6: Figure S6.** Forest plot showing the meta-analysis of hazard ratio estimates for DFS/PFS in all patients. DFS, disease-free survival; PFS, progression-free survival.

**Additional file 7: Table S1.** Results of meta-regression analyses exploring causes of heterogeneity with DFS/PFS in solid tumors.

**Additional file 8: Figure S7.** Forest plots of the association between YKL-40 and clinicopathological parameters. (a)tumor stage (III-IVvs.I-II, C-D vs. A-B or extended vs. limited). Experimental, stage(III-IV, C-D or extended); Control, (I-II, A-B or limited). (b)metastasis status(lymph node or liver metastasis vs. no metastasis). Experimental, lymph node or liver metastasis. Control, no metastasis.

## Data Availability

All relevant data are within the paper and its additional information files.

## Abbreviations

HR: hazard ratio
CIs: confidence intervals
OS: overall survival
TNM: tumor-node-metastasis
hCGP-39: human cartilage glycoprotein-39
CHI3L1: chitinase-3-like-1 protein
mAY: monoclonal anti-YKL-40 antibody
IR: irradiation
DFS: disease-free survival
PFS: progression-free survival
NOS: Newcastle–Ottawa Scale
OR: odds ratio
