# Supplementary figures and images for: Prognostic value of YKL-40 in solid tumors: a meta-analysis of 41 cohort studies
Source: Cancer Cell Int. 2019 Oct 10;19:259. doi: 10.1186/s12935-019-0983-y (PMC6785874; doi:10.1186/s12935-019-0983-y)

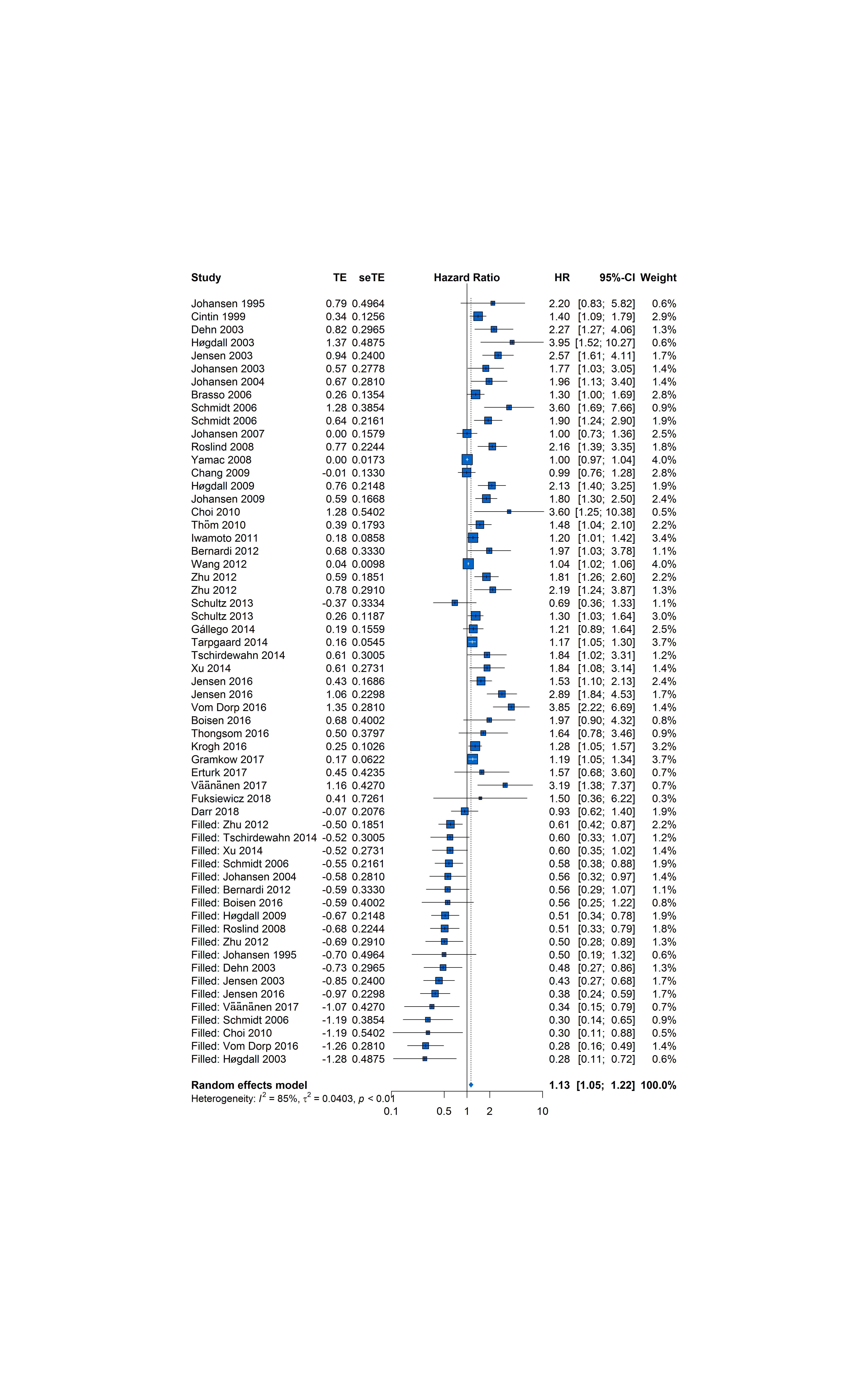

Supplement: Supplementary file 1 — Additional file 1: Figure S1. Forest plot showing the meta-analysis of hazard ratio estimates for overall survival in all patients after the trim-and-fill method was applied. [file 12935_2019_983_MOESM1_ESM.tif]

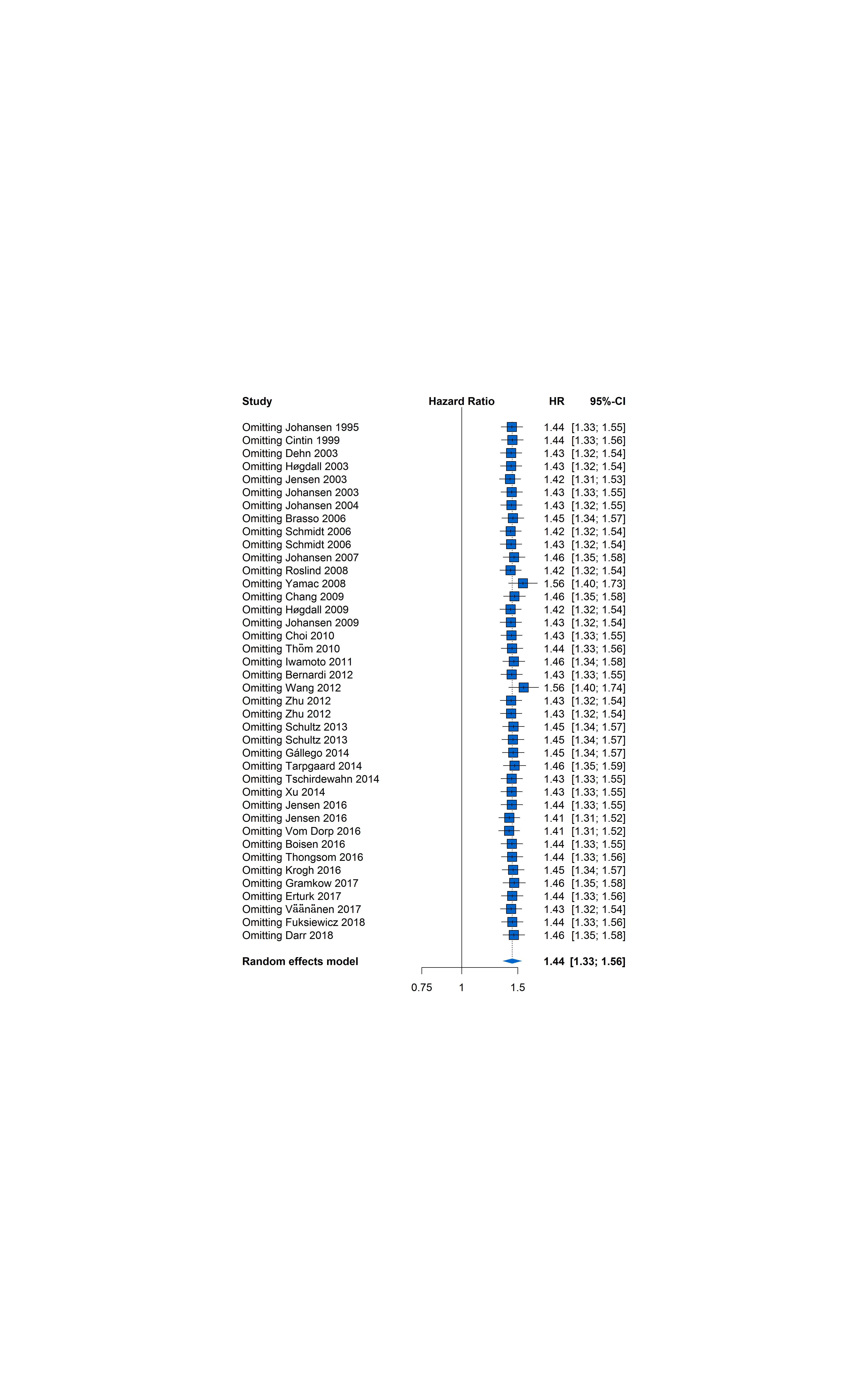

Supplement: Supplementary file 2 — Additional file 2: Figure S2. Sensitivity analysis for the pooled hazard ratios in overall survival in all patients. The analysis was conducted by estimating the average hazard ratio in the absence of each study. [file 12935_2019_983_MOESM2_ESM.tif]

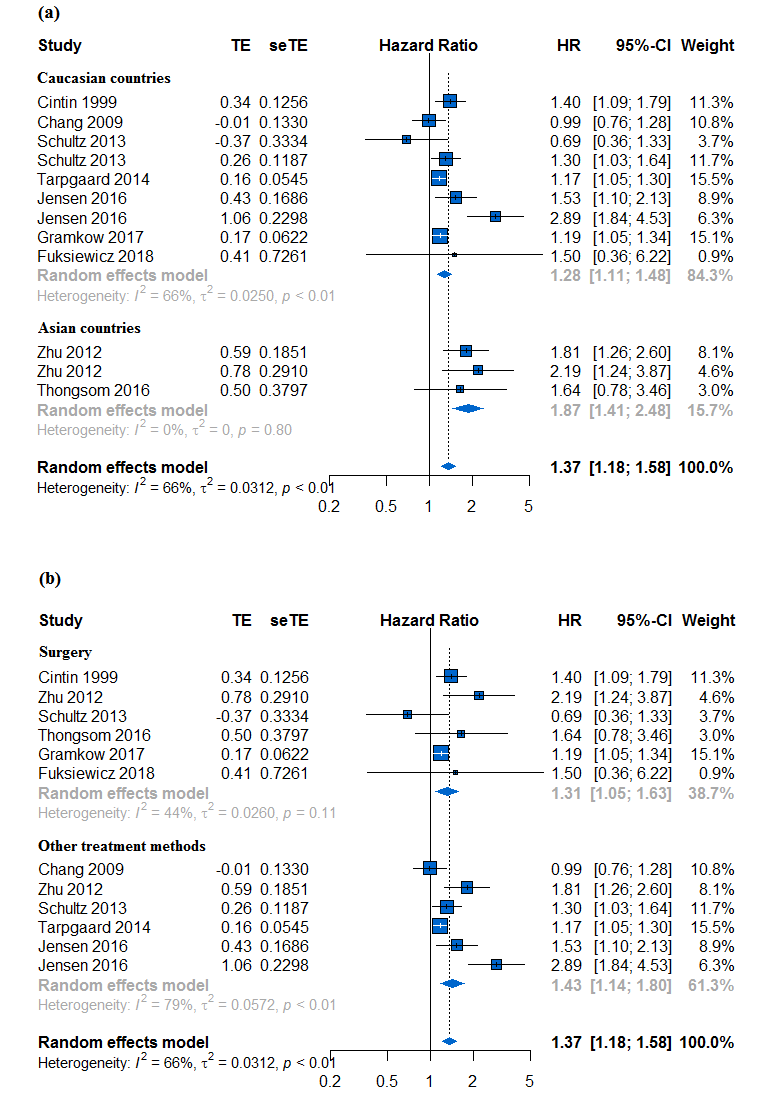

Supplement: Supplementary file 3 — Additional file 3: Figure S3. Forest plot showing the meta-analysis of hazard ratio estimates for overall survival in (a) the “Caucasian countries” subgroup and the “Asian countries” subgroup and (b) the “surgery” subgroup and “other treatment methods” subgroup. [file 12935_2019_983_MOESM3_ESM.tif]

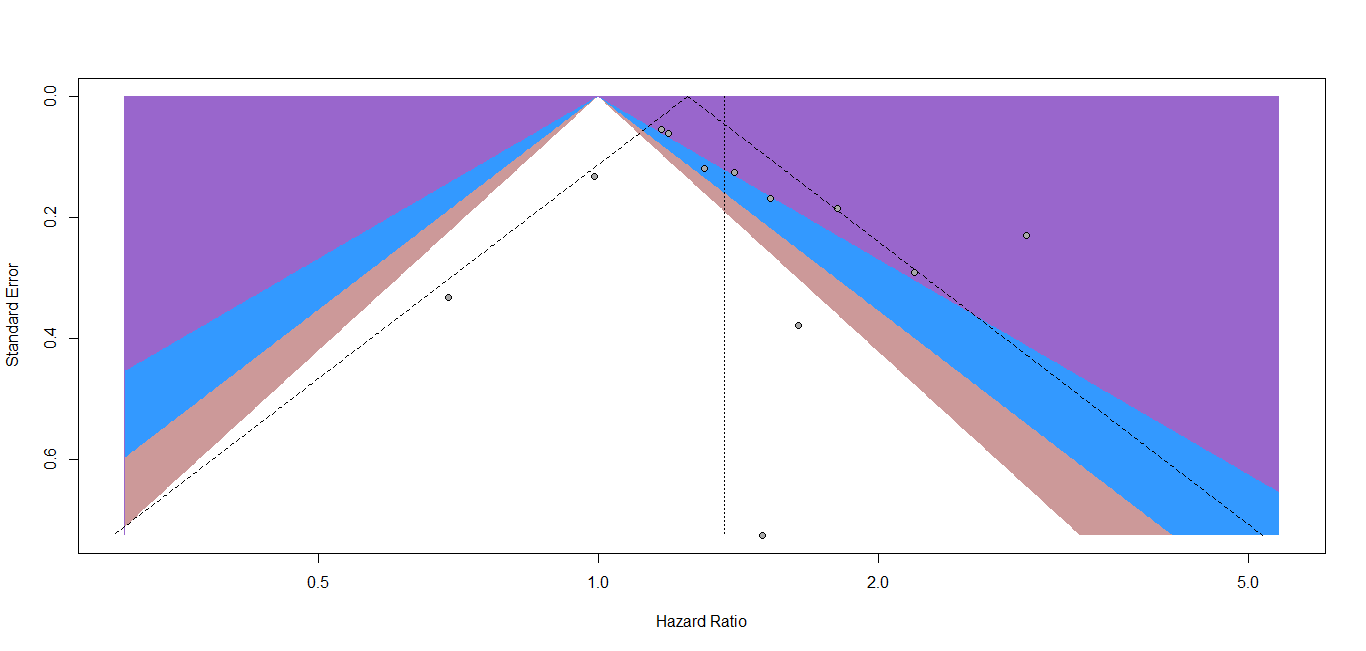

Supplement: Supplementary file 4 — Additional file 4: Figure S4. Contour-enhanced funnel plot of the association between serum/plasma YKL-40 and overall survival in gastrointestinal tumors. [file 12935_2019_983_MOESM4_ESM.tif]

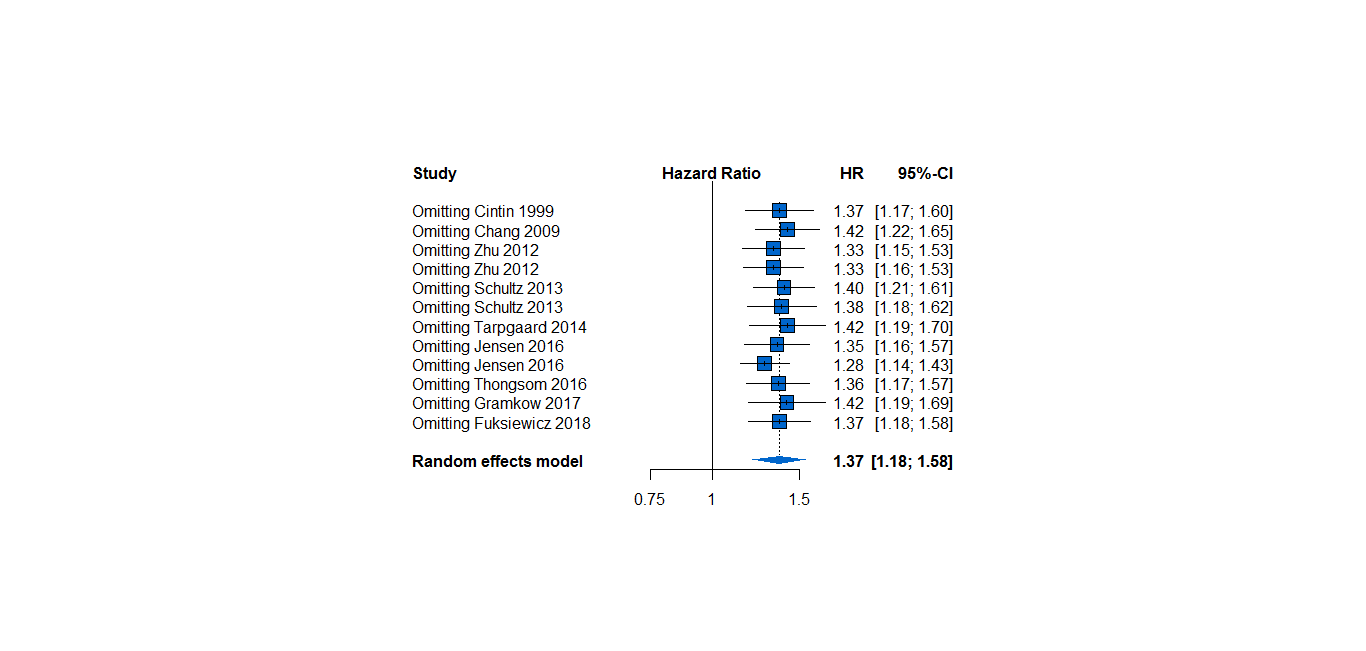

Supplement: Supplementary file 5 — Additional file 5: Figure S5. Sensitivity analysis for the pooled hazard ratios in all patients with gastrointestinal tumors. The analysis was conducted by estimating the average hazard ratio in the absence of each study. [file 12935_2019_983_MOESM5_ESM.tif]

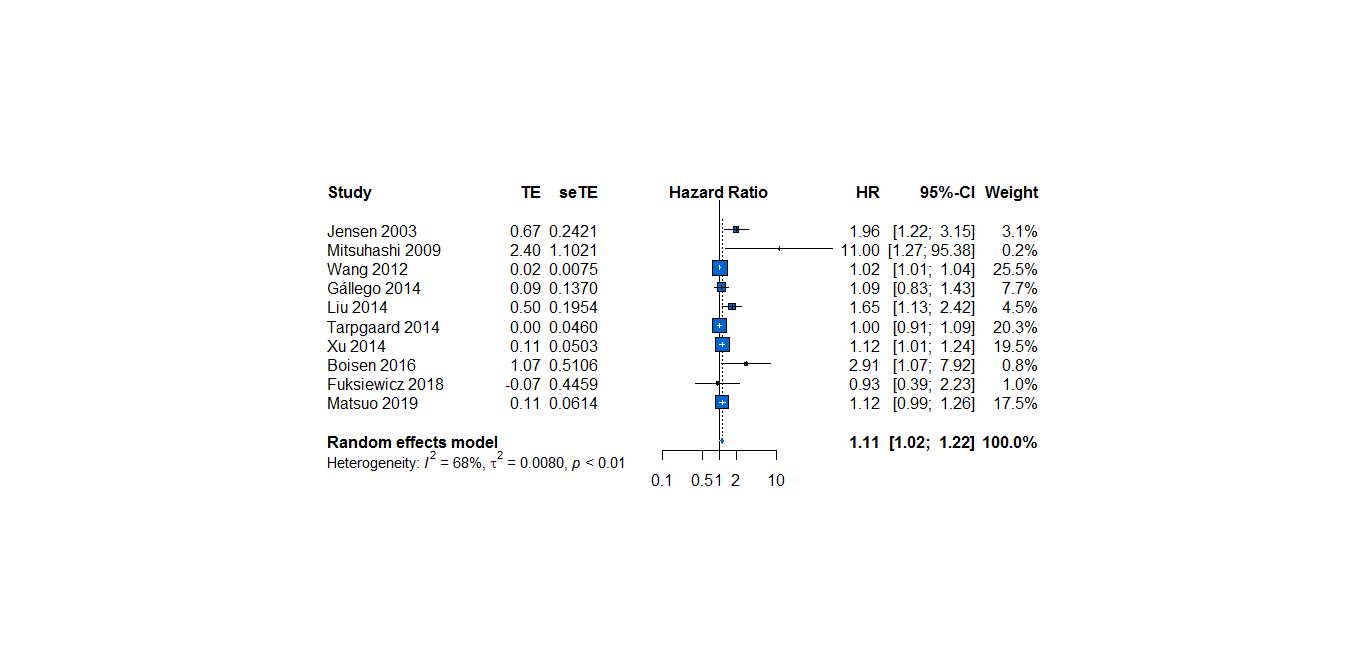

Supplement: Supplementary file 6 — Additional file 6: Figure S6. Forest plot showing the meta-analysis of hazard ratio estimates for DFS/PFS in all patients. DFS, disease-free survival; PFS, progression-free survival. [file 12935_2019_983_MOESM6_ESM.tif]

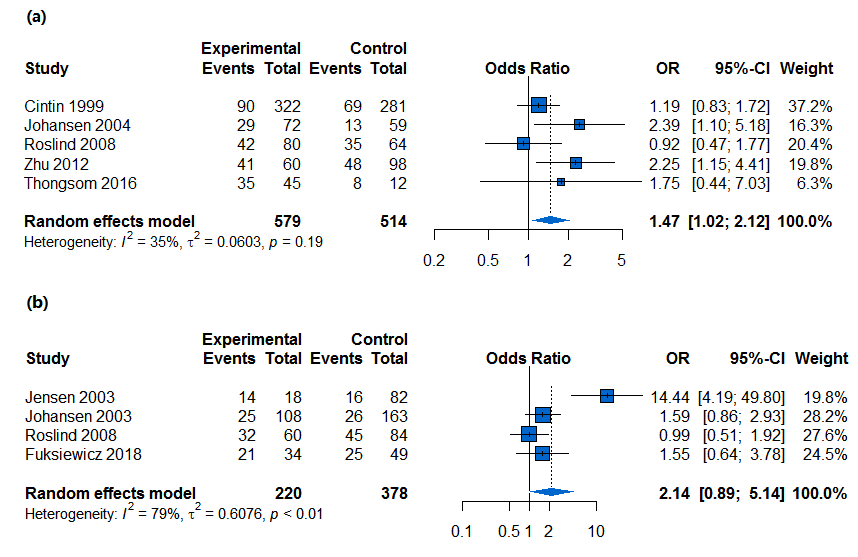

Supplement: Supplementary file 8 — Additional file 8: Figure S7. Forest plots of the association between YKL-40 and clinicopathological parameters. (a)tumor stage (III-IVvs.I-II, C-D vs. A-B or extended vs. limited). Experimental, stage(III-IV, C-D or extended); Control, (I-II, A-B or limited). (b)metastasis status(lymph node or liver metastasis vs. no metastasis). Experimental, lymph node or liver metastasis. Control, no metastasis. [file 12935_2019_983_MOESM8_ESM.tif]
