# Supplementary material for: Prognostic value of YKL-40 in solid tumors: a meta-analysis of 41 cohort studies
Source: Cancer Cell Int. 2019 Oct 10;19:259. doi: 10.1186/s12935-019-0983-y (PMC6785874; doi:10.1186/s12935-019-0983-y)
Supplement: Supplementary file 7 — Additional file 7: Table S1. Results of meta-regression analyses exploring causes of heterogeneity with DFS/PFS in solid tumors. [file 12935_2019_983_MOESM7_ESM.doc]

| **Additional file 7 : Table S1.** Results of meta-regression analyses exploring causes of heterogeneity with DFS/PFS in solid tumors. | |
| --- | --- |
|  | DFS/PFS  Univariate analysis |
| Covariates | P |
| ethnicity | 0.9445 |
| publication year | 0.6929 |
| YKL-40 cutoff value | 0.0017 |
| sample size | 0.0538 |
| proportion of advanced stage | 0.8162 |
| specimen type | 0.4427 |
| treatment method | 0.3932 |
| Note: DFS, disease-free survival, RFS, relapse-free survival, PFS, progression-free survival | |
